# Preparation of Functional Food with Enhanced Antioxidant Properties by Adding *Aronia melanocarpa* Polyphenol Honey

**DOI:** 10.3390/foods13233852

**Published:** 2024-11-28

**Authors:** Jingyi Wang, Jiahui Hao, Jie Wang, Siyu Wang, Ziluan Fan

**Affiliations:** 1 School of Life Sciences, Northeast Forestry University, 26 Hexing Road, Xiangfang District, Harbin 150040, China; owo_guyu@163.com (J.W.); haojiahui5832@163.com (J.H.); wj18365936610@163.com (J.W.); siyuwang1122@163.com (S.W.); 2 Key Laboratory of Forest Food Resources Utilization, Harbin 150040, China

**Keywords:** *Aronia melanocarpa*, honey, antioxidant activity, polyphenols, polyphenol-enriched honey

## Abstract

To enhance the functionality of honey, particularly its antioxidant capacity, the incorporation of *Aronia melanocarpa* polyphenols (AMPs) is an effective approach. The preparation technology and antioxidant activity of AMP added to honey were studied. AMP was extracted with ethanol and its components were analyzed and then mixed evenly with honey in different addition amounts (0.1~0.5% *w*/*w*). The product was characterized based on the active ingredients (total phenols, total flavonoids, and anthocyanin content) and antioxidant activity (DPPH, ABTS, and reducing power) during storage to obtain the optimal storage time. The optimal polyphenol addition amount was determined by combining honey enzyme activity (amylase, glucose oxidase, and sucrase), sensory evaluation, and acute cell toxicity experiments. The optimal preparation process is an addition of 0.4% AMP and a storage time of 14 days or more. The active ingredients of the product are positively correlated with the AMP addition, and the antioxidant activity is significantly improved (from two to eight times). AMP exhibits a notable inhibitory effect on enzyme activity, with concentrations ranging from 0.1% to 0.4%, resulting in enzyme activity levels in honey remaining at 75% or higher. Honey samples containing 0.1% to 0.5% AMP exhibit minimal to no acute toxicity to cells. AMP can improve the nutritional value of honey, imparting unique color and flavor while enhancing its antioxidant activity. As such, it holds significant potential as a novel functional food.

## 1. Introduction

Honey is a natural food extracted by bees from nectar, containing nutrients such as carbohydrates, enzymes, phenols, vitamins, and other compounds, which contribute to its high nutritional value. Its composition is complex, consisting primarily of sugars (80–85%) and water (15–17%), along with trace amounts of enzymes, organic acids, vitamins, proteins, minerals, and phenolic compounds. These components play a crucial role in influencing its sensory and functional properties [1]. The components in honey are influenced by factors such as plant origin, geographical origin, environment and climate, bee species and colony status, processing and storage conditions, etc. Different honey varieties exhibit distinct nutritional profiles and characteristics [2]. Based on their botanical origin, honeys can be classified into two main types: monofloral honey, which is derived from the nectar or honeydew of a single plant species, and polyfloral honey, which is sourced from multiple plant species [3]. Historically, honey has been used in traditional medicine for its antibacterial, antioxidant, anti-tumor, anti-inflammatory, and antiviral properties, among others [4]. In addition, its high osmotic pressure and acidic content contribute to its therapeutic efficacy in treating infectious wounds and burns and promoting healing [5]. However, the production and application of traditional honey have been relatively limited, failing to fully explore its potential added value. With the growing awareness of health and the increasing demand for functional foods, there is a rising need for innovative honey products.

*Aronia melanocarpa*, a member of the Rosaceae family, originated in North America and was transferred to Europe approximately a century ago [6]. *Aronia melanocarpa* is recognized for its exceptionally high anthocyanin content, a class of potent antioxidants [7]. Compared to other fruits within the Rosaceae family, such as strawberries, apples, or pears, Aronia stands out due to its higher concentration of anthocyanins, which significantly enhances its antioxidant potential [8]. Beyond anthocyanins, Aronia also contains substantial amounts of other polyphenolic compounds, including proanthocyanidins and flavonoids, further contributing to its nutritional value [9]. However, the fresh and unprocessed fruits of *Aronia melanocarpa* are rarely consumed due to their bitter taste. In recent years, this fruit has gained popularity among European and American consumers because of its high polyphenol content and high antioxidant activity. Compared to developed countries like the United States, the actual utilization rate of *Aronia melanocarpa* resources in China remains relatively low. Due to its relatively recent introduction, the domestic *Aronia melanocarpa* industry is still in its infancy, with a few integrated enterprises spanning planting, processing, and sales. The industry also faces limited product categories and underdeveloped new product functions, as well as nascent product research and development, resulting in an essentially undeveloped market [10]. The development and utilization of *Aronia melanocarpa* resources have garnered significant attention. Some related enterprises have been established in eastern China, most of which are small and medium-sized agricultural enterprises with limited risk resilience. Since 2014, 18% of enterprises in the sector have ceased operations, reflecting a significant industry contraction [10].

Honey is renowned for its high nutritional value and beneficial properties for human health. Its antioxidant characteristics are primarily attributed to the presence of polyphenolic compounds [11]. Compared to propolis, which contains approximately 5% organic compounds, honey contains only 0.5% organic substances, resulting in relatively lower antioxidant activity [12]. Honey remains one of the most widely consumed bee products; it is hoped to improve its health benefits, including its antioxidant properties. The incorporation of various fruits, herbs, and spices into honey is a relatively novel concept in the beekeeping market. These combinations produce unique flavors, aromas, and visual characteristics. More importantly, they combine the beneficial health properties of honey with those of the added ingredients. Numerous examples of such products exist, some of which have been evaluated for their chemical properties and bioactivity. However, research on these aspects remains limited. Dżugan [13] investigated the addition of natural herbs such as nettle, hawthorn, pine, cranberry, and aloe vera to honey, examining their physicochemical properties and antioxidant activity. The results indicated that the physicochemical properties remained consistent with natural honey; however, a significant increase was observed in phenolic compounds and antioxidant capacity. After adding 1.5% (*w*/*w*) plant extract, the polyphenol content increased by up to six times compared to the control sample (multifloral honey), and the antioxidant activity was enhanced by up to five times. In a separate study, the addition of blackberry and raspberry fruits (1% and 4%) and leaves (0.5% and 1%) to canola honey was investigated. The results demonstrated a significant improvement in the honey’s antioxidant activity, with a four-fold increase observed for the 4% fruit addition and a five-fold increase for the 1% leaf addition [14]. Similarly, Tomczyk [15] enhanced honey by incorporating 4% (*w*/*v*) mulberry leaves and mulberry fruit, resulting in a nearly four-fold increase in polyphenol content and a more than fifty-fold improvement in antioxidant activity. In the study conducted by Tomczyk [15] on the preparation of fortified honey, samples were analyzed after 30 days of storage; however, literature on this topic remains limited. This study aims to refine the processes within the 30-day storage period to optimize production techniques and improve efficiency, providing a reference for future developments. 

**Figure 1:**
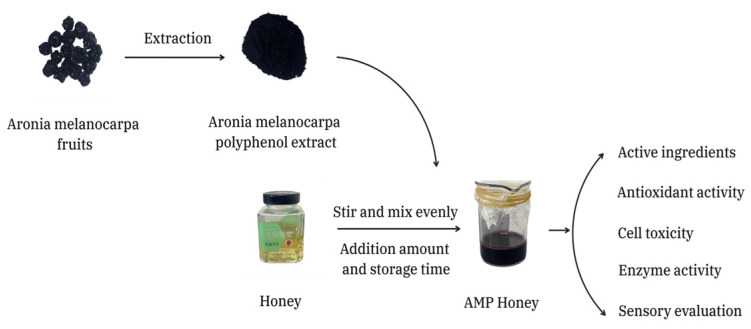
The total experimental contents.

This study is dedicated to incorporating AMP into honey and investigating its optimal preparation technology (Figure 1), aiming to enhance the bioavailability of *Aronia melanocarpa* and enrich the functionality of honey.

The addition of AMP imparts a unique fruity taste to honey, potentially attracting target consumers. This not only broadens the application scope of *Aronia melanocarpa*, but also presents new possibilities for incorporating other natural active substances.

## 2. Materials and Methods

### 2.1. Materials

The plant material (*Aronia melanocarpa*) was collected from the Greater Khingan Range in Heilongjiang Province and stored in a −20 °C freezer in the laboratory. Honey was purchased from a supermarket in Harbin, Heilongjiang Province, and kept at room temperature. HepG2 liver cancer cells were obtained from Gefan Biotechnology (Shanghai, China).

The DMEM high-glucose medium and FBS were purchased from Solarbio (Beijing, China) and Tianhang Biotechnology (Zhejiang, China), respectively, while all other reagents were of analytical grade.

### 2.2. Extraction and Purification of Polyphenols from Aronia melanocarpa

Weigh 200 g of frozen berries on a scale and add 95% ethanol (1:15 *w*/*v*). Homogenize for 5 min using a tissue masher (RZ-8012, Midea, Foshan, Guangdong, China), followed by 3 min using a high-speed homogenizer (BME 100 L, Weiyu, Shanghai, China), and then filter the mixture. Collect filtrate, transfer it in a rotary evaporator, and evaporate at 40 °C to obtain the extract. Dry the extract at 35 °C for 24 h to yield the crude extract of *Aronia melanocarpa* polyphenols.

According to Reference [16], before chromatography, the adsorption material must be pretreated. The specific steps are as follows: Add an appropriate quantity of X-5 macroporous resin to a beaker and soak it in 95% ethanol (*v*/*v*) for 24 h. Subsequently, rinse the resin with deionized water until the alcohol odor is completely removed. Next, soak the resin in a 5% NaOH solution for 12 h, then wash with deionized water until a neutral pH is achieved. After soaking in 10% acetic acid solution for 12 h, wash with deionized water until neutral. The macroporous resin is loaded into the chromatographic column via wet loading. The purification conditions are as follows: loading concentration of 200 mg/mL, elution with 60% ethanol (*v*/*v*), and a flow rate of 1 mL/min. Collect the eluate, concentrate it under reduced pressure, and dry at 35 °C for 24 h to yield the polyphenol extract of *Aronia melanocarpa* (AMP).

### 2.3. Characterization of AMP by UPLC-TQ-MS

A quantity of 1.0 mg of *Aronia melanocarpa* polyphenol extract was weighed and dissolved in mass spectrometry grade methanol to prepare a 1000 µg/mL solution. After centrifugation at 12,000 rpm for 10 min, the solution was filtered through a 0.22 µm filter, and the sample was stored at −20 °C for later use.

UPLC-MS/MS (Vanquis, Thermo Fisher Scientific, Waltham, MA, USA) was employed to identify and analyze the compounds in the polyphenol extract of *Aronia melanocarpa*. A Hypersil Gold column (100 × 2.1 mm, 1.9 µm; Thermo Fisher Scientific, Waltham, MA, USA) was used for separation. Mobile phase A consisted of a 0.1% formic acid aqueous solution, while mobile phase B was methanol. The reverse-phase mobile phase included 5 mM ammonium acetate in an aqueous solution (pH 9, adjusted with ammonia), and mobile phase B was methanol. A gradient elution method was applied with a flow rate of 0.35 mL/min. The experimental data were statistically analyzed using Compound Discoverer software (version 3.2, Compound Discoverer, Waltham, MA, USA), which provided accurate information on relative molecular weights and molecular fragments. Based on a comparison with the relevant literature, the chemical structure of important active components in *Aronia melanocarpa* were deduced, enabling qualitative analysis.

### 2.4. Preparation of AMP Honey Addition

We weighed 50 g of honey and added AMP at concentrations of 0.1%, 0.2%, 0.3%, 0.4%, and 0.5% (*w*/*w*). The mixture was stirred using a magnetic stirrer and kept at room temperature, protected from light. The optimal storage time was determined by measuring the active ingredients and antioxidant activity at 7, 14, 21, and 28 days. The amount of AMP added was selected based on its effects on enzyme activity in honey, sensory evaluation, and cytotoxicity. The optimal level of AMP was determined by evaluating its effects on honey enzyme activity, sensory properties, and cytotoxicity. We assessed the potential inhibitory effects on enzyme activity, establishing a safe addition range. Sensory tests ensured that AMP did not alter honey’s flavor, vital for market acceptance. Cytotoxicity testing confirmed no harm to cells or human health. These factors guided the selection of the ideal AMP level to maximize antioxidant benefits while preserving honey quality and safety.

### 2.5. Total Phenolic Content (TPC)

The total phenol content was quantified using the Folin–Ciocalteu colorimetric method [17]. Specifically, 2.5 mL of 0.2 mol/L Folin–Ciocalteu reagent was combined with 0.5 mL of 2% sample solution, mixed thoroughly, then allowed to stand for 5 min. Subsequently, 2 mL of 0.1 g/mL Na_2_CO_3_ solution was added, and the final volume was adjusted to 10 mL in a volumetric flask. The mixture was kept in the dark for 2 h before measuring the absorbance at 725 nm using a spectrophotometer (EPOCH2, Biotek, Winooski, VT, USA), with distilled water serving as the control. Results were reported as milligrams of gallic acid per gram of fresh *Aronia melanocarpa* fruit (mg·g^−1^).

### 2.6. Total Flavonoids Content (TFC)

Referring to the method of Chen [18], TFC was determined using the sodium nitrite-aluminum nitrate method. A 0.16 mL aliquot of the sample solution was transferred into a 2 mL centrifuge tube. To this, 0.32 mL of water and 0.08 mL of a 5% NaNO_2_ solution were added and mixed thoroughly. The mixture was allowed to stand for 6 min before 0.08 mL of a 10% aluminum nitrate solution was added. After shaking well, the solution was left to stand for an additional 6 min. Next, 0.80 mL of a 4% NaOH solution was added, the volume was adjusted with water, and the solution was mixed again. The solution was then allowed to stand for 15 min. The absorbance was measured at a wavelength of 510 nm, with methanol serving as the blank control. The results were expressed in grams per kilogram of fresh fruit (g·kg^−1^).

### 2.7. Total Anthocyanin Content (TAC)

The total monomeric anthocyanin content was determined using the pH differential method [19]. One milliliter of the extract was transferred to two separate volumetric flasks. To one flask, a KCl solution at pH 1 was added, while a CH_3_COO^−^ solution at pH 4.5 was added to the other. The mixtures were incubated at room temperature for 30 min. Absorbance at 510 nm and 700 nm was subsequently measured for both samples. The results were expressed as milligrams of cyanidin-3-glucoside (C3G) per gram of sample. Each analysis was performed in triplicate.

(1) $$A={\left(A_{510}-A_{700}\right)}_{\mathrm{pH}1.0}-{\left(A_{510}-A_{700}\right)}_{\mathrm{pH}4.5}$$

(2) $$\mathrm{TAC}\;(\mathrm{mg}/100g)=\frac{A\times \mathrm{MW}\times \mathrm{DF}\times V}{\varepsilon \times L\times M_{t}}\times 100$$

where A—sample absorbance; MW—molecular weight (449.2 g/mol for cyanidin-3-glucoside); DF—dilution factor; V—total extract volume (mL); ε—molar absorptivity of cyan0idin 3-glucoside(26,900 dm^3^/mol × cm); M_t_—sample mass (g); L—optical path (1 cm).

The total anthocyanin content in honey samples was determined according to the method described by Miłek [20]. A 2 g quantity of honey is diluted in 10 mL of 0.1% HCl ethanol solution and incubate at 60 °C for 1 h, with occasional shaking. The solution is then filtered through filter paper and the volume is adjusted to 10 mL in a volumetric flask using acidified ethanol. The absorbance of the extract is measured at 657 and 530 nm using a spectrophotometer (EPOCH2, Biotek, Winooski, VT, USA), and the net absorbance is calculated using Formula (3).

(3) $$A=A_{530}-0.25\;\times \;A_{657}$$

The anthocyanin content in the honey samples is determined using Formula (2).

### 2.8. Antioxidant Activity Assay

#### 2.8.1. DPPH Radical Scavenging Assay

The reference was slightly modified [21]. A 1.0 mL aliquot of the sample was mixed with 1.0 mL of 0.2 mmol/L DPPH solution and incubated in the dark for 30 min. The absorbance was then measured at 517 nm using absolute ethanol as the blank, with the value recorded as A_1_. The absorbance of distilled water, replacing the sample, was measured and recorded as A_3_. The absorbance of absolute ethanol, replacing the DPPH solution, was measured and recorded as A_2_. The DPPH radical scavenging capacity of the samples is calculated using Formula (4), with ascorbic acid as a positive control.

(4) $$\mathrm{DPPH}\;\mathrm{scavenged}\;(\%)=(1-\frac{A_{1}-A_{2}}{A_{3}})\;\times \;100$$

where A_1_ is the absorbance value of the experimental group; A_3_ is the absorbance value of the control group; and A_2_ is the absorbance value of the blank group.

#### 2.8.2. ABTS Radical Scavenging Assay

The procedure was slightly modified [21]. A 7 mmol/L ABTS solution and a 2.45 mmol/L K_2_S_2_O_8_ solution were prepared separately and mixed in equal volumes. The mixture was incubated at room temperature in the dark for 12 to 16 h. Subsequently, 1.0 mL of the sample was combined with 3 mL of the ABTS working solution. Distilled water was used as the blank, and for the control, absolute ethanol was substituted for the ABTS working solution. The mixture was then incubated in the dark at room temperature for 30 min. The absorbance was measured at 734 nm, with the value recorded as A_4_. The absorbance of distilled water, replacing the sample, was measured and recorded as A_6_. The absorbance of absolute ethanol, replacing the ABTS solution, was measured and recorded as A_5_. The scavenging ability of the samples against ABTS radicals is calculated using Formula (5), with ascorbic acid as the positive control.

(5) $$\mathrm{ABTS}\;\mathrm{scavenged}\;(\%)=(1-\frac{A_{4}{-A}_{5}}{A_{6}})\times 100,$$

where A_4_ is the absorbance value of the experimental group; A_6_ is the absorbance value of the control group; and A_5_ is the absorbance value of the blank group.

#### 2.8.3. Reducing Power Assay

The reference was slightly modified [21]. To prepare the test solution, 1 mL of the sample was combined with 2.5 mL of PBS buffer (0.2 mol/L, pH 6.6) and 2.5 mL of a 1% potassium ferrocyanide solution. The mixture was then heated in a water bath at 50 °C for 20 min. Afterward, 2.5 mL of 10% trichloroacetic acid was added, followed by 2.5 mL of distilled water and 0.5 mL of 0.1% ferric chloride solution. The mixture was thoroughly mixed and allowed to stand for 10 min. The absorbance was measured at 700 nm, with distilled water used for blank calibration.

### 2.9. Effects on the Viability of HepG2 Liver Cancer Cells

#### 2.9.1. Culture of HepG2 Cells

(1) Cell Recovery

The HepG2 cell cryovial was removed from the −80 °C freezer and rapidly thawed at 37 °C. The thawed cells were transferred to a 10 mL sterile centrifuge tube and mixed with 6 mL of DMEM culture medium. The mixture was centrifuged (H/T20MM, Herexi, Changsha, Hunan, China) at 1000 rpm for 5 min, and the supernatant was discarded. A volume of complete medium (DMEM supplemented with 1% antibiotics and 10% FBS) was added, and the cells were resuspended gently. The cell suspension was then transferred to a culture flask, which was placed in a 37 °C incubator (246, Memmer, Hofstrach, SN, Germany) with 5% CO_2_ for 24 h, during which the medium was replaced to maintain cell health.

(2) Cell Passaging

Cell passage is performed when the cell density reaches 80~90% confluence in the culture flask. First, the old medium is removed, and the flask is rinsed twice with 2 mL of PBS. Next, 1 mL of trypsin is added for digestion, and the process is monitored under a microscope (DMi8, Sartorius, Gottingen, NI, Germany). When most cells become rounded and detached, digestion is halted. The trypsin is removed, and an appropriate volume of complete medium is added. The flask is gently tapped to detach and disperse the cells into a single-cell suspension. Half of the suspension is transferred to a new culture flask, and fresh medium is added to both flasks and mixed. The flasks are then disinfected and placed in a 37 °C incubator with 5% CO_2_. The culture medium is regularly changed over the next 1~2 days while closely monitoring cell growth.

(3) Cell Cryopreservation

Cell freezing is performed during the logarithmic growth phase. First, the old culture medium is removed, and the flask is rinsed twice with 2 mL of PBS. Then, 1 mL of trypsin is added for cell digestion, and the process is monitored under a microscope. Once the majority of cells have rounded up and detached, digestion is halted. The trypsin is removed, and an appropriate volume of culture medium is added. The flask is gently tapped to dislodge the cells. The cell suspension is then transferred to a 10 mL centrifuge tube and centrifuged at 1000 rpm for 5 min to remove the supernatant. The freezing solution (FBS: DMSO = 9:1) is added, and the cells are gently mixed to ensure uniform distribution. The mixture is transferred to cryovials, placed at 4 °C for 30 min, then at −20 °C for 30 min, and finally stored at −80 °C for long-term preservation.

#### 2.9.2. Determination of HepG2 Cell Viability

This study employed the MTT assay to evaluate the impact of samples on the viability of HepG2 liver cancer cells, assessing their cytotoxicity. HepG2 cells were inoculated into a 96-well plate at a density of 5.0 × 10^4^ cells/mL (100 µL per well). To prevent evaporation, 200 µL of PBS was added to the outer wells. After disinfection with 75% ethanol, the plate was incubated at 37 °C in a 5% CO_2_ environment for 24 h. Various concentrations of polyphenol solutions (0, 20, 40, 60, 80, 100, 120 µg/mL) were then prepared and added (100 µL per well) for an additional 24 h of incubation. Subsequently, 10 µL of MTT was added, and the plate was incubated at 37 °C for 4 h. After the supernatant was discarded, 100 µL of DMSO was added, and the plate was shaken for 10 min to dissolve the formazan crystals. Absorbance was measured at 560 nm, and cell viability was expressed as the ratio of the absorbance of treated cells to that of the untreated control. Each experiment was performed in triplicate, with six replicates for each treatment condition and control.

### 2.10. Determination of the Effect on Enzyme Activity in Honey

#### 2.10.1. Sucrose Invertase Assay

The determination of sucrose-converting enzyme activity follows the method described by Wang [22]. Transfer 1 g of honey into a 10 mL volumetric flask. Dissolve 10 g of sucrose in a small volume of water, and transfer the solution to a 100 mL volumetric flask. Add 10 mL of pH 6.8 phosphate buffer solution, and dilute to the mark with water to prepare the sucrose solution. Mix 5 mL of the honey dilution with 5 mL of the sucrose solution, then transfer 2.5 mL of the mixture to a 100 mL volumetric flask and dilute to the mark. Incubate the sample at 45 °C for 1 h. Before and after the conversion, take 1 mL of the sample, add 2 mL of 3,5-dinitrosalicylic acid, heat in a water bath for 3 min, cool, and dilute to 25 mL. Measure the absorbance at 540 nm. Analyze the reducing sugar content before and after conversion using a standard curve and calculate the sucrose invertase activity: sucrose invertase activity (mg/g·h) = 10 × 2 (monosaccharide content after conversion, monosaccharide content before conversion). With glucose as the standard, a standard linear regression equation was drawn (y = 1.8716x + 0.0092; R^2^ = 0.9999), and the sucrose invertase activity in the honey sample was calculated in mg/g·h using the reference equation.

#### 2.10.2. Glucose Oxidase Assay

Glucose oxidase activity was determined using Tian’s method [23]. The sample solution was incubated in a water bath at 37 °C for 30 min, after which 4 mL of the sample was aliquoted. In a 25 mL stoppered test tube, 3 mL of acetate buffer (pH 3.5) and 1.3 mL of a 1 mmol/L indigo carmine solution were added. The solution was then diluted to the 25 mL mark and mixed thoroughly. The mixture was heated in a boiling water bath for 13 min, cooled in running water for 5 min, and its absorbance was measured at 612 nm. The glucose oxidase activity in the honey samples was calculated using the standard linear regression equation (y = 0.0105x + 0.0033; R^2^ = 0.9996), and the results were expressed in units of µg/g·0.5 h.

#### 2.10.3. Amylase Assay

The determination of amylase activity was conducted following the method described by Yi [24]. A 5 g sample was accurately weighed and mixed with 2.5 mL of acetate-acetic acid buffer, 1.5 mL of 0.5 mol/L sodium chloride solution, and 10 mL of distilled water. The mixture was dissolved, transferred to a 25 mL volumetric flask, and diluted to the mark. The resulting solution was heated in a water bath to 40 °C. Then, 10 mL of the honey solution, maintained at the same temperature, was mixed with 5 mL of gelatinized starch solution. After thorough mixing, the timer was started, and absorbance was measured at 660 nm every 5 min until the absorbance dropped below 0.235. The time (t, in minutes) required for the absorbance to decrease to 0.235 was recorded. The amylase activity of the honey was then calculated using the formula DN = 300/t, where amylase activity is expressed in units of mL/(g·h).

### 2.11. Sensory Evaluation

#### 2.11.1. Determination of Solubility

The method described by Wei was followed with slight modifications [25]. A 5 g sample was weighed and mixed with 37.5 mL of hot water at 60 °C. The solubility of the sample was observed.

#### 2.11.2. Determination of Pfund Value

Reference for experimental methods [26]. Prepare a 50% honey aqueous solution (*w*/*v*), gently heat it to 50 °C to completely dissolve the crystals, measure the absorbance at a wavelength of 635 nm, and convert the result to the mm Pfund value according to Formula (6).

mm Pfund = −38.70 + (371.39 × Abs),(6)

#### 2.11.3. Sensory Evaluation

The analysis was conducted in the Sensory Analysis Laboratory at the School of Life Sciences, Northeast Forestry University (NEFU). A total of 20 evaluators (10 male and 10 female students) from NEFU were recruited to form the sensory evaluation panel. The participants had an average age of 22 ± 2 years and underwent preliminary training before the evaluation process.

The evaluators were required to be in good health, with normal sensory perception, and free from any history of allergies or medication that could affect sensory sensitivity. Before the evaluation, they were instructed to cleanse their mouths 30 min in advance and avoid consuming strongly flavored or spicy foods. Smoking and snacking (including coffee) were prohibited during the evaluation. Evaluators were also asked not to bring any external odors, such as tobacco or cosmetics, that could interfere with the assessments of others. The evaluations were conducted at least two hours after a main meal, with each sample (20 g at 25 ± 2 °C) served in a transparent cup. Evaluators were required to rinse their mouths with purified water before tasting each sample. After tasting one sample, a 10 min break was observed before moving on to the next. Each evaluator assessed all samples in triplicate.

Based on the sensory quality requirements for honey outlined in the literature [27,28,29] and considering the characteristics of the product, sensory evaluation of the AMP honey products was conducted in four main categories: appearance, color, aroma, and taste. All evaluators used the criteria outlined in Table 1 to score five AMP honey products on a 100-point scale. Appearance and color each contributed 20 points, while aroma and taste each accounted for 30 points. Higher scores indicated better quality in each respective attribute. Due to the naturally bitter berry-like taste of the *Aronia melanocarpa*, particular emphasis was placed on assessing the impact of AMP on the aroma and taste of the honey.

All experimental protocols were approved by the Animal Care and Use Committee of Northeast Forestry University (Harbin, Heilongjiang Province, China), under ethical approval number IACUC-2024041 (6 March 2024).

### 2.12. Statistical Analysis

All experiments were conducted in triplicate, and all tests were performed at least three times. Variance analysis was carried out using SPSS software (version 25.0, SPSS, Chicago, IL, USA), and the Duncan method (*p* < 0.05) was employed to assess the significance of differences between experimental values. Data processing and chart generation were performed using Excel (version 2016, Excel, Redmond, WA, USA) and Origin (version 2018, Origin, Northampton, MA, USA).

## 3. Results

### 3.1. Component Analysis of AMP by UPLC-TQ-MS

**Figure 2:**
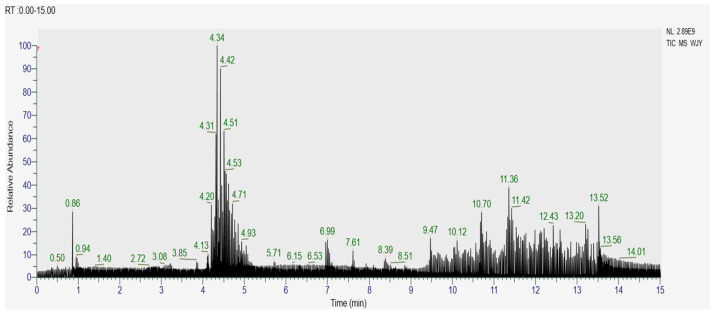
Total ion flow chart of polyphenol extract from *Aronia melanocarpa*.

The active ingredients in AMP were analyzed by UPLC-TQ-MS. The total ion chromatogram (Figure 2) and the 16 identified compounds (Table 2) are presented.

According to Table 2, AMP contains four types of anthocyanins, all of which belong to the cornflower family (*m*/*z* 287). Peak 1 exhibits a quasi-molecular ion peak at [M+H]+449.10541, with a fragment ion at *m*/*z* 287, corresponding to the characteristic mass-to-charge ratio of cyanidin-3-glucoside. Previous studies [30,31] have shown that the fragment ion *m*/*z* 287 results from the loss of a hexose molecule from the parent ion, forming [M-162]+. By correlating the mass spectrometry data with information from databases such as MzCloud, MzVault, ChemSpider, and MassBank, Peak 1 is identified as cyanidin-3-glucoside. Similarly, peaks 2 through 4 correspond to cyanidin-3-O-arabinoside, cyanidin-3-galactoside, and cyanidin-3-O-xyloside, respectively. Previous research [32] has shown that the main anthocyanins in *Aronia melanocarpa* include cyanidin-3-galactoside, cyanidin-3-O-arabinoside, cyanidin-3-glucoside, and cyanidin-3-O-xyloside, which is consistent with the findings of the present study.

Table 2 shows that AMP contains eight flavonoids. The quasi-molecular ion peak at *m*/*z* 287.05383 (peak 5) is accompanied by two fragment ions at *m*/*z* 241 and 213. The fragment ion at *m*/*z* 241 is formed by the loss of CH_2_O_2_ from the parent ion, resulting in the ion [M+H-46]+, while the fragment ion at *m*/*z* 213 is formed by the loss of C_2_H_4_ from the *m*/*z* 241 ion, resulting in [M+H-28]+. Based on the mass spectrometry data from databases such as mzCloud, mzVault, ChemSpider, and MassBank, it can be concluded that peak 5 corresponds to kaempferol.

The quasi-molecular ion peak detected at *m*/*z* [M+H]+ 303.04919 in peak 6 is accompanied by three fragment ions at *m*/*z* 285, 257, and 229. According to the literature analysis [33], the fragment ion at *m*/*z* 285 corresponds to the quasi-molecular ion [M+H]+ at *m*/*z* 303, which is formed by quercetin losing a water molecule (H_2_O) from the A ring under 40% energy bombardment. The fragment ion at *m*/*z* 257 results from the loss of CO from the C ring of the fragment ion at *m*/*z* 285, while the fragment ion at *m*/*z* 229 is generated from the rearrangement and CO loss of the *m*/*z* 257 fragment. This fragmentation pattern is consistent with that of the standard quercetin. In summary, based on mass spectrometry data from databases such as mzCloud, mzVault, ChemSpider, and MassBank, it can be inferred that peak 6 corresponds to quercetin.

The quasi-molecular ion peaks at [M-H]- 433.20703 and [M-H]- 433.07681 for peaks 7 and 8 are observed, with three fragment ions at *m*/*z* 300, 255, and 136. According to previous studies [34], a parent ion corresponding to *m*/*z* 300 [M+H-132]- is observed, resulting from the cleavage of pentose. A fragment ion at *m*/*z* 255 is generated by the loss of a CHO_2_ molecule, suggesting that peaks 7 and 8 are derivatives of quercetin-pentose. Based on the quasi-molecular ion peaks and mass spectrometry data from databases such as MzCloud, MzVault, ChemSpider, and MassBank, peak 7 is identified as quercetin-3-D-xyloside, and peak 8 as quercetin-3-arabinoside.

The quasi-molecular ion peak detected in peak 9 is [M-H]- 463.08563, and a secondary fragment ion at *m*/*z* 301 is generated, corresponding to [M+H-162]-. According to the literature [35], the *m*/*z* 301 fragment arises from the loss of a glucose residue in peak 9. Additional fragment ions observed at *m*/*z* 255, 227, and 151 correspond to those of standard quercetin. Therefore, peak 9 is identified as a derivatized flavonoid glycoside of quercetin. Based on the *m*/*z* 301 fragment ion, which results from the loss of a glucose residue, it is speculated to be quercetin-3β-D-glucoside. This is further supported by mass spectrometry data from databases such as mzCloud, mzVault, ChemSpider, and MassBank, which suggest that peak 9 is quercetin-3β-D-glucoside.

The quasi-molecular ion peak detected in peak 10 is [M-H]- 595.12933, and there is a fragment ion *m*/*z* 301, which corresponds to the characteristic mass-to-charge ratio of quercetin. It is speculated that peak 10 represents a flavonoid glycoside with quercetin as the aglycone. A 294 Da molecule is lost between the two main fragment ions, *m*/*z* 595 and *m*/*z* 301, which corresponds to a disaccharide loss. According to the literature [36], it is inferred that the compound is a quercetin-3-O-sangbu disaccharide. Combining the mass spectrometry data from databases such as mzCloud, mzVault, ChemSpider, and MassBank, it can be concluded that peak 10 is a quercetin-3-O-sangbu disaccharide.

The quasi-molecular ion peak detected in peak 11 is [M-H]- 289.07138, and there is a fragment ion *m*/*z* 137, which is the characteristic mass-to-charge ratio of catechins. In negative ion mode, in addition to the molecular ion peak *m*/*z* 289, the RDA reaction also occurs to generate the typical secondary fragment *m*/*z* 137 (1, 3A) of flavanol flavonoids, and a fragment *m*/*z* 245 is generated by losing a molecule of CO_2_. According to the literature [37], it is inferred that they are catechins. Combined with mass spectrometric data from databases such as mzCloud, mzVault, ChemSpider, and MassBank, it can be determined that peak 11 corresponds to catechins.

The quasi-molecular ion peak detected in peak 12 is [M-H]- 609.14508, which is associated with two fragment ions of *m*/*z* 301 and 255. According to the literature analysis [38], the *m*/*z* 301 fragment is derived from the loss of rutinose following collision-induced dissociation under 25% energy bombardment, while the *m*/*z* 255 fragment results from the loss of the C-ring carbonyl group after the *m*/*z* 303 fragment undergoes cleavage. This fragmentation pattern is consistent with the standard rutin fragment ion profile. In conclusion, based on mass spectrometry data from databases such as MzCloud, MzVault, ChemSpider, and MassBank, it can be inferred that peak 12 represents rutin.

Table 2 shows that AMP contains four phenolic acid compounds. The quasi-molecular ion peaks detected in peaks 13, 14, and 15 are [M-H]- 353.08636, [M-H]- 353.08710, and [M-H]- 353.08643, respectively. A parent ion is observed at *m*/*z* 353 [M-H]- in the MS spectrum. In the MS/MS spectrum, characteristic chlorogenic acid fragments are observed at *m*/*z* 191 and *m*/*z* 135 [39]. Based on mass spectrometry data from databases such as mzCloud, mzVault, ChemSpider, and MassBank, it can be inferred that peaks 13 and 14 correspond to chlorogenic acid, while peak 15 corresponds to neochlorogenic acid.

The quasi-molecular ion peak detected in peak 16 is [M+H]+ 171.99263, with fragment ions at *m*/*z* 154, 131, 108, and 97, which are characteristic of gallic acid. Based on the mass spectrum data provided by databases such as mzCloud, mzVault, ChemSpider, and MassBank, it is suggested that peak 16 corresponds to gallic acid.

The quasi-molecular ion peak detected in peak 17 is [M+H-H_2_O]+ 163.03850, with fragment ions at *m*/*z* 135, 117, and 89, which are characteristic of caffeic acid. Based on the mass spectrum data provided by databases such as mzCloud, mzVault, ChemSpider, and MassBank, it is suggested that peak 17 corresponds to caffeic acid.

### 3.2. Active Ingredients

**Figure 3:**
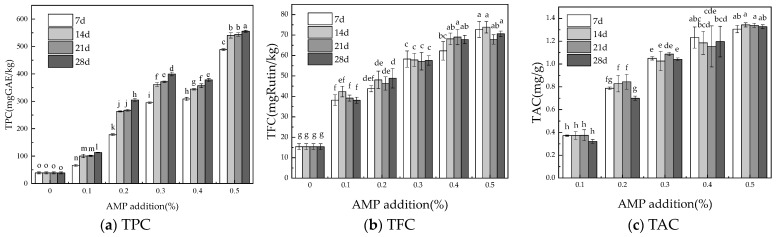
Changes in active ingredients of AMP honey during storage. Changes in AMP honey compared to original honey over 7, 14, 21, and 28 days regarding (**a**) TPC; (**b**) TFC; (**c**) TAC. GAE (gallic acid equivalent); TPC (total phenolic content); TFC (total flavonoid content); TAC (total anthocyanin content). The results are expressed as mean ± standard deviation (n = 3). Different lowercase letters indicate significant differences (*p* < 0.05).

The changes in active ingredients of honey products supplemented with added AMP during storage were assessed, and the results are shown in Figure 3.

Phenolic compounds are characterized by their aromatic ring structure and hydroxyl groups, which act as electron donors and directly promote antioxidant activity [40]. In addition, some phenolic compounds stimulate the synthesis of endogenous molecules within cells. They exhibit several mechanisms of action, including free radical inhibition, peroxide decomposition, metal inactivation, and deoxygenation [41]. These antioxidants also display a range of biological activities, such as anti-inflammatory, anti-atherosclerotic, and anticancer effects, likely due to their antioxidant properties [42]. In this study, the total phenolic content (TPC) in *Aronia melanocarpa* was 152.29 ± 2.73 mg GAE/g FW. Cong [43] measured 11 batches of *Aronia melanocarpa* fruits and found that the TPC ranged from 174.685 ± 1.17 to 190.998 ± 1.56 mg/g, which is similar to our results. In a recent study on *Aronia melanocarpa*, Denev [44] reported the TPC of *Aronia melanocarpa* extract to be 7849 mg/100 g DW. The difference in TPC between our results and those of Denev may be attributed to variations in the origin, growth conditions, climatic factors, storage methods, and other variables affecting the *Aronia melanocarpa* samples. Figure 3a illustrates the TPC of AMP honey during storage, showing a significant increase in TPC with AMP addition. Over time, TPC exhibited an overall upward trend, with a notable increase observed from 7 to 14 days of storage. The TPC of samples with AMP additions of 0.2%, 0.3%, and 0.4% did not differ significantly, whereas the TPC of samples with 0.5% AMP addition was the highest, reaching 555.64 mg GAE/kg.

Flavonoids are abundant in both edible and medicinal plants, typically occurring as glycosides and possessing multiple phenolic hydroxyl groups. Their free radical scavenging activity is particularly effective due to the presence of hydroxyl groups in various positions and the ortho-dihydroxy structure on the B ring [45]. Flavonoids are widely found in foods and beverages commonly consumed, such as fruits, vegetables, tea, chocolate, and wine [46]. Research suggests that flavonoids may lower the risk of several health conditions, including type 2 diabetes, cardiovascular disease, obesity, and non-alcoholic fatty liver disease [47]. In this study, the total flavonoid content (TFC) of *Aronia melanocarpa* was 50.35 ± 0.14 mg/kg FW. Figure 3b shows the TFC of AMP honey during the storage period. As illustrated, the TFC of honey increased significantly after the incorporation of AMP. With increasing AMP concentration, the TFC of AMP honey exhibited a gradual upward trend. The TFC of AMP was 50.35 ± 0.14 mg/kg FW, while the TFC of honey was 15.39 ± 1.45 mg/kg. At an AMP concentration of 0.3%, the TFC ranged from 57.16 to 58.26 mg/kg, higher than the individual TFC values of AMP and honey, suggesting a synergistic effect. Over time, the TFC initially increased, followed by a decline. Notably, there was a marked increase in TFC between days 7 and 14 of storage, likely due to the instability and subsequent degradation of flavonoids.

In this study, the total anthocyanin content (TAC) in *Aronia melanocarpa* was determined using the pH differential method. The experiment was repeated three times, and the average TAC value was 164.65 3.22 mg/100 g FW. The increase in anthocyanin concentration during the ripening of *Aronia melanocarpa* enhanced its color and visual appeal [48]. Denev [49] found that TAC in different sources of *Aronia melanocarpa* ranged from 428 to 1790 mg/100 g FW. Subsequently, Denev [50] analyzed the composition of *Aronia melanocarpa* berries from various growers in Bulgaria. Although all berries belonged to the same variety and were harvested during the same season, the total anthocyanin content (TAC) exhibited variability, ranging from 284 to 686 mg/100 g fresh weight (FW). TAC varies in different *Aronia melanocarpa* due to factors such as variety, fertilization conditions, maturity, harvest time, and planting area. Figure 3c shows the TAC of honey with AMP added during storage. As storage time increases, TAC generally shows a slow decrease, likely due to the decomposition of anthocyanins during storage. As the amount of AMP added increases, TAC shows an upward trend. A significant upward trend is observed between 0.1% and 0.2% AMP addition. However, the TAC of samples with 0.3% to 0.5% AMP addition does not increase significantly. The sample with 0.5% AMP addition has the highest TAC, measuring 1.34 mg/g.

There is a dose–response relationship between the active ingredients in AMP honey products and the amount of AMP added. However, when the addition is between 0.4% and 0.5%, there is no significant increase in TFC and TAC content, which may be due to the low water content of honey and the limited solubility of AMP. TPC increases with storage time, while TFC and TAC exhibit an overall trend of initially increasing and then gradually decreasing with prolonged storage, possibly due to the unstable degradation of flavonoids and anthocyanins.

### 3.3. Antioxidant Activity

Oxidation is a fundamental metabolic process in all organisms. Free radicals generated during oxidation are a primary cause of cellular damage. An imbalance between the production of reactive oxygen and nitrogen species (ROS/RNS) and the activity of antioxidant enzymes leads to oxidative stress. This oxidative damage harms tissues and biological molecules, such as lipids, DNA, and proteins, impairing their ability to defend against further oxidative damage. Moreover, exposure to ROS and resulting oxidative stress significantly contribute to the onset of chronic diseases, such as inflammation, diabetes, cancer, neurodegeneration, and cardiovascular disease [51]. Antioxidants can be used to counteract oxidative stress [52]. These substances are categorized as either endogenous or exogenous. Antioxidants work by scavenging free radicals, chelating metal ions, and inhibiting pro-oxidative enzymes. In vitro evaluation methods for antioxidant activity are favored due to their simplicity and speed, while exogenous antioxidants from dietary sources play a crucial role in mitigating oxidative stress in the human body [53]. There is a strong correlation between antioxidants, their activity, and the methods used to assess their capacity. To achieve a comprehensive evaluation, it is essential to establish a systematic assessment framework. A single in vitro method has limitations, so combining multiple methods provides a more objective evaluation of antioxidant capacity.

#### 3.3.1. DPPH Radical Scavenging Assay

**Figure 4:**
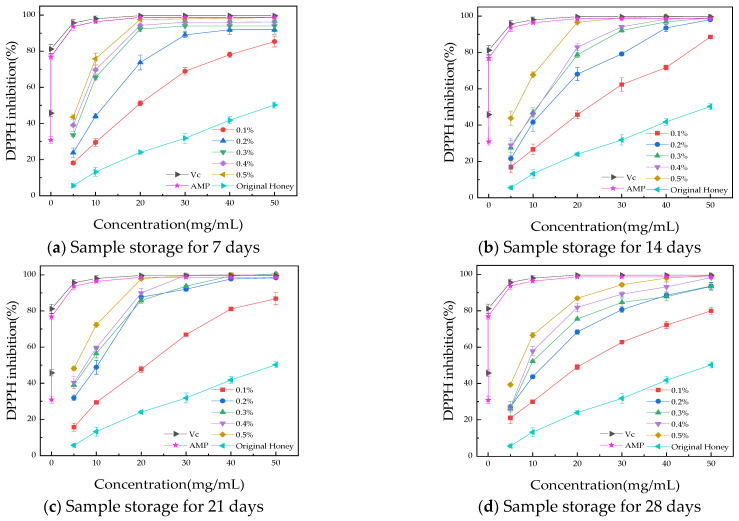
The DPPH free radical clearance rate during the storage period. (**a**) Sample storage for 7 days; (**b**) sample storage for 14 days; (**c**) sample storage for 21 days; (**d**) sample storage for 28 days.

To evaluate the antioxidant activity of AMP honey products during storage, the DPPH free radical scavenging rate was measured, with Vc used as a control. Figure 4 illustrates that both AMP honey products and the original honey exhibit antioxidant activity, with the former showing a stronger DPPH free radical scavenging ability observed at higher sample concentrations. A positive correlation was observed between the concentration of the samples and the rate of DPPH free radical scavenging. The scavenging activity of AMP against DPPH free radicals increased in a dose-dependent manner with higher concentrations of AMP. As the AMP content increases, the scavenging ability of the samples against DPPH free radicals surpasses that of the original honey. This could be attributed to the exceptionally high antioxidant capacity of AMP, which enhances the DPPH free radical scavenging ability of honey. As storage time increased, the DPPH free radical scavenging ability of the samples improved, with stronger oxidation resistance observed at 21 days. This may be due to the enhanced stability of the AMP honey system over time. After 28 days of storage, the IC_50_ values for the original honey and AMP-enriched honey samples (0.1%, 0.2%, 0.3%, 0.4%, and 0.5% AMP) were 50.61 ± 2.00 mg/mL, 21.01 ± 0.49 mg/mL, 12.81 ± 0.14 mg/mL, 11.03 ± 0.55 mg/mL, 9.80 ± 0.52 mg/mL, and 6.31 ± 0.33 mg/mL, respectively. Compared to the original honey, the antioxidant activity was enhanced by a factor of 2 (0.1% AMP) to 8 (0.5% AMP) following the addition of AMP.

#### 3.3.2. ABTS Radical Scavenging Assay

**Figure 5:**
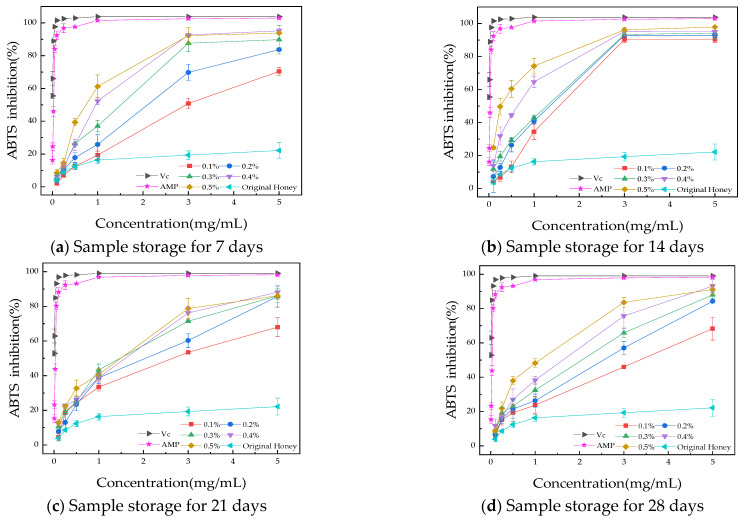
The ABTS free radical clearance rate during the storage period. (**a**) Sample storage for 7 days; (**b**) sample storage for 14 days; (**c**) sample storage for 21 days; (**d**) sample storage for 28 days. The results are presented as mean value ± standard deviation (SD; n = 3).

Figure 5 shows the ABTS free radical scavenging ability of the honey product with AMP added during storage. Both the honey product with AMP and the original honey exhibit antioxidant capacity. As the sample concentration increases, so does the ABTS scavenging ability, confirming a positive correlation between concentration and scavenging rate. AMP demonstrated significant antioxidant activity, and as its concentration increased, the ABTS scavenging rate also increased, showing a clear dose–response relationship. As AMP content increases, the sample demonstrates a stronger ability to scavenge ABTS free radicals, which is significantly greater than that of the original honey. This may be due to AMP’s high antioxidant capacity, which enhances the honey’s ABTS free radical scavenging ability. Over time, the sample’s ability to scavenge ABTS free radicals improves, reaching a peak at 14 days and stabilizing by 21 days. After 28 days of storage, at a sample concentration of 5 mg/mL, the free radical scavenging rates of honey and AMP-enriched honey (0.1%, 0.2%, 0.3%, 0.4%, and 0.5% AMP) for the ABTS assay were 22.18 ± 4.86%, 68.34 ± 6.67%, 84.23 ± 0.70%, 87.97 ± 2.10%, 93.12 ± 3.79%, and 91.10 ± 3.05%, respectively. Compared to the original honey, the antioxidant activity increased by a factor of 3 (0.1% AMP) to 4 (0.4% and 0.5% AMP) following AMP addition.

#### 3.3.3. Reducing Power Assay

**Figure 6:**
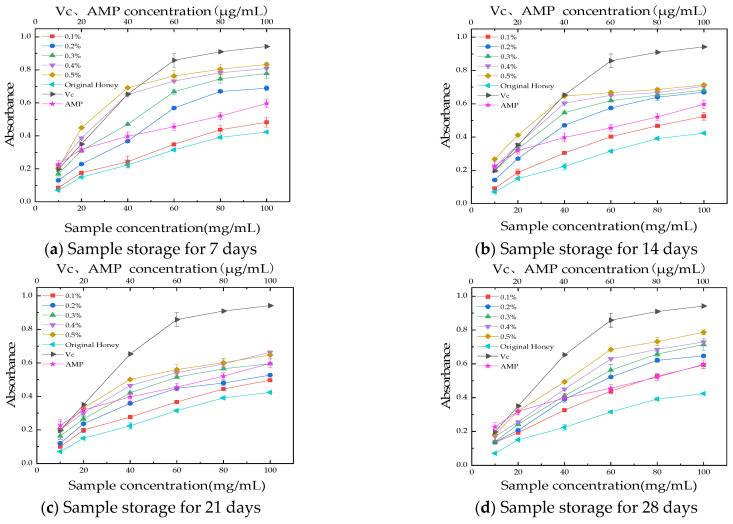
Total reduction power during storage. (**a**) Sample storage for 7 days; (**b**) sample storage for 14 days; (**c**) sample storage for 21 days; (**d**) sample storage for 28 days. The results are presented as mean value ± standard deviation (SD; n = 3).

The determination of reducing power investigates the electron-donating ability of the sample. Samples with high reducing power are typically good electron donors, and the electrons provided by them can not only reduce Fe^3+^ to Fe^2+^ but also react with free radicals, transforming them into stable substances. In the presence of antioxidants, the Fe^3+^/ferrocyanide complex can be reduced to Fe^2+^/ferrocyanide, leading to the formation of a strong Prussian blue complex through the binding of the ligand with Fe^2+^. Consequently, the amount of reduced iron can be quantified by measuring the absorbance of Prussian blue at 700 nm, which correlates with the concentration of antioxidants [54]. The reducing power indicates a significant correlation between the strength of the electron-donating ability and the antioxidant activity, meaning that the magnitude of the reducing power can reflect the strength of the sample’s antioxidant ability [55]. Figure 6 illustrates the reducing power of AMP honey products over the storage period. It can be observed that with an increase in sample concentration, there is a corresponding rise in absorbance values, indicating enhanced antioxidant activity. Additionally, as the amount of AMP incorporated into the samples increases, both the absorbance values and the reducing capacity demonstrate a positive correlation. After being stored for 7 to 21 days, the total reducing power gradually weakened, and after 21 days, the AMP honey system stabilized, showing an increase in its total reducing power.

### 3.4. HepG2 Cell Viability

**Figure 7:**
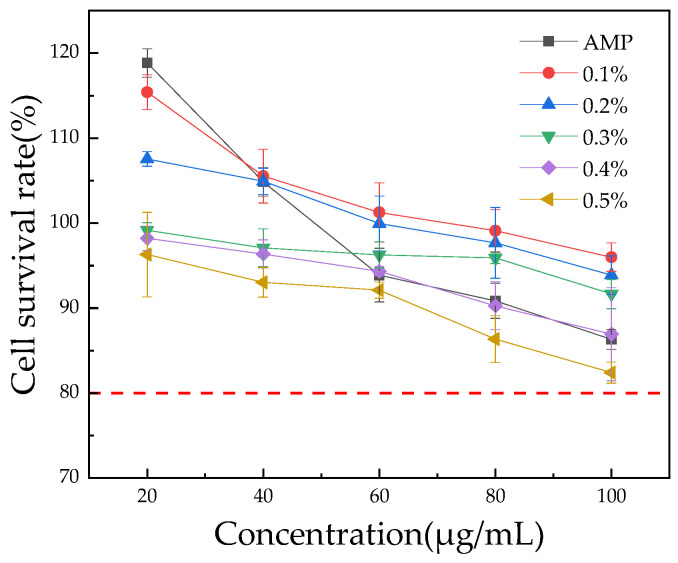
Cytotoxicity assay. The red dashed line represents a cell viability of 80%. The results are expressed as mean ± standard deviation (n = 3).

The HepG2 cell line, which has strong metabolic capacity, provides an in vitro model closest to the human liver, making it ideal for cytotoxicity testing [56]. The cell survival rate of HepG2 cells treated with *Aronia melanocarpa* polyphenols for 24 h was measured to evaluate acute toxicity. The acute toxicity of AMP and honey products, with varying concentrations of AMP, on HepG2 cells after 24 h was assessed using the MTT assay, with the results presented in Figure 7.

As shown in Figure 7, within the concentration range of 5~100 µg/mL, the cell survival rate of HepG2 cells treated with AMP for 24 h was greater than 80%. Therefore, AMP has no significant cytotoxic effect on the cells within this concentration range. In agreement with these findings, Shi [57] observed that *Aronia melanocarpa* polyphenols, when applied to HepG2 cells for 24 h within a concentration range of 10 to 500 µg/mL, resulted in a cell survival rate exceeding 90%, with no acute toxicity observed.

The survival rate of HepG2 cells decreases with the increase of AMP honey product concentration, and the higher the amount added, the lower the cell survival rate. At concentrations ranging from 20 to 100 µg/mL, the 24 h survival rate of HepG2 cells treated with 0.1% to 0.5% AMP honey samples exceeded 80%. Therefore, it can be inferred that honey products exhibit minimal cytotoxicity at these concentrations. Honey products with AMP concentrations of 0.1% to 0.2% were non-toxic to cells at sample concentrations between 20 and 60 µg/mL, with a cell survival rate greater than 100%, peaking at 115.41 ± 1.68%. At a concentration of 100 µg/mL, the survival rates of HepG2 cells treated with honey products containing 0.1%, 0.2%, 0.3%, 0.4%, and 0.5% AMP were 86.33 ± 0.96%, 95.99 ± 1.37%, 93.89 ± 1.85%, 91.66 ± 1.41%, 86.93 ± 4.48%, and 82.41 ± 1.01%, respectively. These results suggest that low concentrations of AMP do not significantly harm cell viability and may even promote cell growth to some extent, while higher concentrations inhibit cell growth. Shi [57] investigated the effects of polyphenols from *Aronia melanocarpa* on the activity of HepG2 cells under oxidative stress, finding that concentrations between 0.01 and 0.05 mg/mL promoted cell growth. Wei [58] studied the protective effects of quercetin on oxidative stress-induced damage in HepG2 cells and found that low concentrations of quercetin exhibited no significant toxicity to HepG2 cells, with a cell survival rate greater than 90%, which was not significantly different from the control group.

### 3.5. Effect on Enzyme Activity in Honey

The main biological enzymes in honey are amylase, glucose oxidase, and sucrase, which are mainly derived from bee saliva and as animal-derived enzymes. Enzyme activity in honey is influenced by factors such as nectar collection time, bee colony age, nectar quantity, and the concentration and composition of nectar [59]. To examine the impact of varying AMP addition levels on honey enzyme activity, we measured the activities of sucrase, glucose oxidase, and amylase, and the results are shown in Table 3.

The amylase enzyme is an important component in honey, and its concentration is primarily influenced by the freshness, processing, and storage conditions. Due to amylase’s sensitivity to temperature and storage conditions, the amylase activity in honey serves as an indicator of its freshness and whether it has undergone heat treatment [60]. The amylase activity of the honey sample was measured at 8.58 ± 0.15 mL/g·h, which complies with the national standard that requires the amylase activity to be no less than 4 mL/g·h. As shown in Table 3, the amylase activity decreases as the AMP concentration increases, although it remains above the national standard. At 0.5% AMP, the amylase value is 5.21 ± 0.26 mL/g·h, reflecting a 39.28% reduction in activity. At 0.4% AMP, the amylase value is 6.58 ± 0.31 mL/g·h, with a 23.31% reduction in activity. When the AMP concentration ranges from 0.1% to 0.4%, amylase activity remains above 75%. Devarajan [61] found that phenolic compounds in honey exert an inhibitory effect on amylase activity, with higher concentrations of nectar leading to greater inhibition.

The activity of glucose oxidase decreased with increasing AMP concentrations, ranging from 83.52 μg/g·0.5h to 57.81 μg/g·0.5h. At an AMP concentration of 0.5%, the glucose oxidase value was 57.81 ± 1.99 μg/g·0.5h, corresponding to a 35.42% decrease in activity. At an AMP concentration of 0.4%, the glucose oxidase value was 67.61 ± 2.34 μg/g·0.5h, with a decrease of 24.48% in activity. When the AMP concentration ranged from 0.1% to 0.4%, the glucose oxidase activity remained above 75%.

Sucrase activity plays a crucial role in honey maturation and can be used as an indicator of its maturity. In this experiment, sucrase activity decreased with increasing AMP concentrations, ranging from 41.84 mg/g·h to 29.47 mg/g·h. Sucrase activity is notably higher than that of amylase, and the magnitude of change is greater. This is because sucrase is more sensitive to environmental factors than amylase [62]. At an AMP concentration of 0.5%, sucrase activity was 29.47 ± 1.09 mg/g·h, corresponding to a 32.63% decrease; at 0.4% AMP, sucrase activity was 33.49 ± 1.47 mg/g·h, with a 23.43% decrease. When the AMP concentration ranged from 0.1% to 0.4%, sucrase activity remained above 75%.

The comprehensive determination of amylase, glucose oxidase, and sucrase activities in honey revealed that the addition of AMP inhibited enzyme activity. The enzyme activity remained at or above 75% when the AMP concentration ranged from 0.1% to 0.4%.

### 3.6. Sensory Evaluation

#### 3.6.1. Solubility

Table 4 shows the solubility properties of honey products with added AMP. It can be observed that the addition of AMP increases the viscosity of honey, possibly due to the decrease in its water content following the dissolution of polyphenols. AMP honey exhibits a distinctive purple color and berry-like aroma, reminiscent of *Aronia melanocarpa*. However, as the amount of AMP added increases, undissolved AMP particles may appear, likely because the low water content of honey limits its ability to dissolve a larger quantity of polyphenols.

#### 3.6.2. Pfund Value

**Figure 8:**
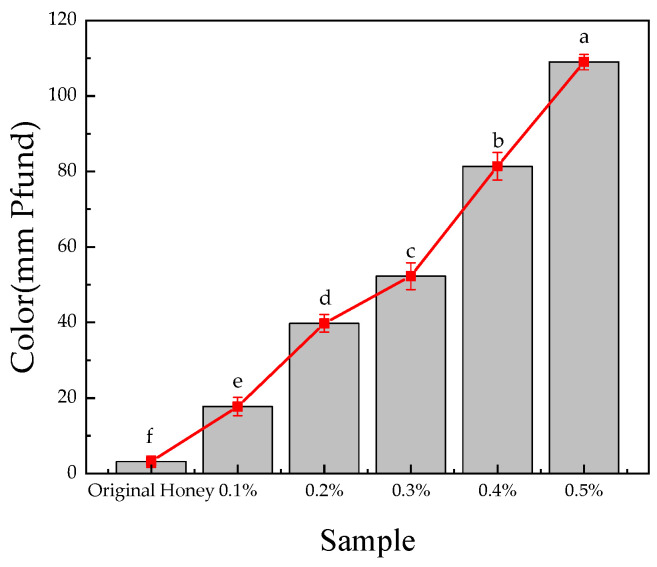
Effect of AMP addition on the color of honey. The results are expressed as mean ± standard deviation (n = 3). Different lowercase letters indicate significant differences (*p* < 0.05).

Color is the most immediate sensory attribute of honey, influenced by various factors, including temperature, storage conditions, and the source of the honey. According to the Pfund grade, the color of honey products from white to dark amber. The color of honey products with different AMP additions is shown in Figure 8. The original color of honey, measured at 3.14 ± 1.50 mm Pfund (≤8), is watery white. The addition of AMP significantly darkened the honey color. AMP is rich in anthocyanins, compounds known for their color-deepening properties. Studies have reported that the total anthocyanin content (TAC) in fresh *Aronia melanocarpa* fruit ranges from 209 to 1019.80 mg/100 g, contributing to its dark purple appearance [63]. As the amount of AMP added increases, the Pfund value gradually increases. When 0.5% AMP is added, the color of the honey product is the darkest, reaching 108.99 ± 2.05 mm Pfund.

#### 3.6.3. Sensory Evaluation

**Figure 9:**
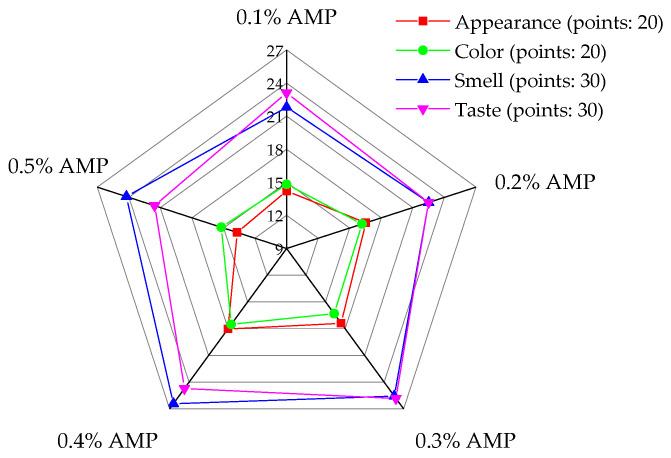
Effect of AMP addition on sensory evaluation of honey.

Sensory evaluation is a critical tool for assessing the quality characteristics of food, playing a key role in determining the authenticity of products [64]. Figure 9 presents the sensory evaluation results of honey products with varying levels of AMP addition.

Figure 9 shows the sensory scores of honey samples with varying concentrations of AMP. The overall sensory scores followed the trend: 0.4% AMP > 0.3% AMP > 0.2% AMP > 0.1% AMP > 0.5% AMP. As the AMP concentration increased from 0.1% to 0.4%, the sensory scores generally improved across all attributes. Specifically, AMP contributed a distinctive berry-like flavor, which enhanced the aroma and taste of the honey. Additionally, the deep purple color of the honey, when AMP was added within an appropriate concentration range, received higher ratings for appearance.

At lower concentrations (e.g., 0.1%), the honey exhibited a less pronounced berry aroma, and its color and appearance were less appealing. This suggests that a higher AMP concentration is necessary to achieve a noticeable impact on the sensory characteristics. Conversely, at the 0.5% concentration, the honey exhibited visible AMP particles, negatively affecting its appearance. Additionally, the flavor was overshadowed by a strong astringency, masking the natural taste of the honey and resulting in an overall poorer sensory experience. 

The optimal sensory qualities in terms of color, texture, and aroma were observed when the AMP concentration ranged from 0.3% to 0.4%. These samples received the highest sensory scores for color, organization, and aroma, indicating that this concentration range is ideal for enhancing the sensory appeal of honey without compromising its overall quality. In conclusion, while AMP has a positive effect on the sensory attributes of honey, careful attention to its concentration is critical to achieving the best balance of flavor, appearance, and texture.

## 4. Discussion

The health industry represents a core sector in the 21st century, reflecting a societal shift towards promoting healthy lifestyles. This paradigm emphasizes not merely the “treatment of diseases,” but, more importantly, the “prevention of diseases before they occur.” The focus is on eliminating suboptimal health, enhancing physical fitness, reducing discomfort, and effectively managing health through protection and maintenance. This transition marks a movement from a passive consumption model that prioritizes treatment to an active consumption model that emphasizes prevention. 

With the ongoing development of the food industry and an increasing emphasis on health, the market for functional foods is expanding. Numerous studies indicate that the consumption of polyphenol-rich foods can significantly benefit health and lower the risk of chronic diseases. *Aronia melanocarpa*, known for its abundant resources and wide cultivation range, is rich in nutrients and possesses various physiological functions. However, its sour and astringent taste makes it unsuitable for fresh consumption, which severely limits its development and utilization. Therefore, there is a pressing need for deep processing or extraction of its natural active compounds for further development.

This study successfully developed a honey product enriched with polyphenols derived from *Aronia melanocarpa*, assessing its biological activity, sensory properties, and acute cytotoxicity to determine the optimal preparation process. In comparison to traditional honey, AMP honey exhibits a captivating purple-red color and the berry aroma characteristic of *Aronia melanocarpa*, alongside enhanced antioxidant activity. The antioxidant active ingredients in this product help protect the primary and secondary metabolites of food from oxidation, rancidity, and degradation, thereby preserving food quality. Moreover, dietary antioxidants can exert beneficial effects on the body, further promoting overall health.

In future research, there is significant potential to explore the synergistic effects of various natural plant active compounds in combination with different honey sources, thereby enriching the diversity of honey categories. The composition of honey and natural plants from the same species can vary significantly across different regions. Additionally, it is worthwhile to explore the variations that may arise from their combinations or formulations. A particularly intriguing avenue is the utilization of syrup infused with natural plant active ingredients to regulate bee feeding, which could facilitate the incorporation of ideal plant bioactive compounds into honey. The degree of enrichment of honey is contingent upon the specific plant additives employed, as distinct plant species generate unique secondary metabolites that may exhibit varying biological activities. For instance, *Aronia melanocarpa* possesses a range of beneficial biological activities and can serve as a model for investigating its enrichment within AMP honey products, meriting further molecular-level study. Future studies could extend the storage period to investigate the stability and quality changes of honey over time further, gradually addressing the long-term effects of storage duration on honey quality. Additionally, this research can be expanded to assess the efficacy and safety of AMP honey products in vivo, thereby providing valuable insights for prospective clinical applications.

From a practical perspective, the market for honey products is characterized by a substantial demand; however, the available offerings tend to be homogeneous. In light of the current market gap and evolving consumer trends toward honey and health products, AMP honey products should consider adopting a product differentiation strategy. By implementing robust positioning techniques and targeting niche markets, these products can circumvent competition with traditional honey manufacturers, thereby establishing a strong foothold within the intensely competitive honey market through differentiated product and marketing strategies. Compared to other health products, AMP honey exhibits considerable market vitality and vast potential. It can be utilized not only as a sweetener but also as a nutritional health product, a culinary ingredient, and for its medicinal properties. Improvements in purification technologies, optimization of production processes, and innovations in modern processing techniques aimed at enhancing product stability and extending shelf life will further facilitate the application of natural products in honey. Additionally, it is essential to consider the factors related to honey product labeling and regulatory compliance. In this context, product labeling and compliance should be adjusted in accordance with the relevant laws and regulations of the respective countries or regions.

In summary, this study establishes a foundational framework for the incorporation of natural plant active ingredients into honey, emphasizing its potential to enhance bioavailability and antioxidant activity. Continued research and development are essential to fully uncover the possibilities for the formulation of this product and its tangible benefits for human health.

## 5. Conclusions

Polyphenols extracted from *Aronia melanocarpa* are incorporated into honey, combining its benefits with the health-promoting properties of the polyphenols. This combination alters the bioactivity of the honey in a dose-dependent manner, significantly enhancing its antioxidant potential.

The low polyphenol content in honey is significantly increased by the addition of AMP, which enhances anthocyanins, a potent antioxidant component. The antioxidant activity of the honey–polyphenol mixture can be influenced by antioxidant methods, polyphenol types, mixture concentration, polyphenol concentration, and storage time. AMP reduces the activity of natural enzymes in honey; when the addition is between 0.1% and 0.4%, the activity remains at 75% or higher. The solubility and color of honey are affected by *Aronia melanocarpa*. As the polyphenol content increases, the viscosity of the mixture becomes thicker, and the color becomes darker. The sensory score is higher when the dosage is 0.3~0.4%. As the polyphenol content increases, the viscosity of the mixture becomes thicker, and the color becomes darker. AMP honey is a promising functional food with enhanced antioxidant potential.

## Figures and Tables

**Table 1:** Sensory evaluation criteria.

Index	Score (Points)	Evaluation Project
Appearance (20 points)	16~20	At room temperature, the product is in paste form, with crystalline solids possibly present. AMP powder may also be observed and uniformly distributed.
	11~15	The product is in a paste state at room temperature, with crystalline solids potentially present. AMP is uniformly distributed throughout.
	<11	The product is in paste form at room temperature, with some or all crystalline solids potentially present. AMP is unevenly distributed.
Color (20 points)	16~20	It has a deep purple color characteristic of AMP and is uniformly distributed.
	11~15	The color is pale, and the distribution is more uniform.
	<11	The color is too light, and the distribution is uneven.
Smell (30 points)	21~30	It has the aroma of nectar-producing flowers, with the inclusion of *Aronia melanocarpa*, and lacks any undesirable odor.
	11~20	The honey flavor and *Aronia melanocarpa* fruit flavor are strong or light, without any undesirable odor.
	<11	The honey flavor and the *Aronia melanocarpa* fruit flavor are too strong or too weak, accompanied by a distinct off-putting odor.
Taste (30 points)	21~30	It has a viscous mouthfeel, a distinct berry flavor, and a rich taste.
	11~20	It has a thick, sticky mouthfeel, a subtle berry flavor, and a rough flavor.
	<11	There is an absence of sticky mouthfeel, berry flavor, and bitter or sour flavor, along with a rough texture.

**Table 2:** The content of major phenolic compounds in *Aronia melanocarpa*.

Peak No.	Chemical Name	Formula	Exact Mass (*m*/*z*)	RT (min)	Characteristic MS/MS Ions (*m*/*z*)
1	Cyanidin-3- glucoside	C_21_H_21_O_11_	449.10541 [M+H]+1	4.236	287.05408
2	Cyanidin-3- O-arabinoside	C_20_H_19_O_10_	419.09497 [M+H]+1	4.318	287.05396
3	Cyanidin-3- galactoside	C_21_H_21_O_11_	449.16202 [M+H]+1	3.439	287.05490
4	Cyanidin-3- galactoside	C_20_H_19_O_10_	418.38831 [M+H]+1	8.172	287.05353
5	Kaempferol	C_15_H_10_O_6_	287.05383 [M+H]+1	4.289	241.04889, 213.05402
6	Quercetin	C_15_H_10_O_7_	303.04919 [M+H]+1	4.880	285.02867, 257.04352, 229.04895
7	Quercetin-3-D- xyloside	C_20_H_18_O_11_	433.20703 [M-H]−1	5.066	300.02686, 255.02942, 136.35802
8	Quercetin-3- arabinoside	C_20_H_18_O_11_	433.07681 [M-H]−1	5.066	300.02686, 255.02942, 136.35802
9	Quercetin-3β- D-glucoside	C_21_H_20_O_12_	463.08563 [M-H]−1	4.840	301.03455, 255.02928, 227.03438, 151.00238
10	Quercetin-3-O-sangbu disaccharide	C_26_H_28_O_16_	595.12933 [M-H]−1	4.671	301.03452, 255.02934
11	Catechins	C_15_H_14_O_6_	289.07138 [M-H]−1	4.557	245.07950, 136.50128
12	Rutin	C_27_H_30_O_16_	609.14508 [M-H]−1	4.784	301.0388, 255.02943
13	Chlorogenic acid	C_16_H_18_O_9_	353.08636 [M-H]−1	4.390	191.05478, 135.04340
14	Chlorogenic acid	C_16_H_18_O_9_	353.08636 [M-H]−1	4.582	191.05478, 135.04340
15	Neochlorogenic acid	C_16_H_18_O_9_	353.08643 [M-H]−1	4.132	191.05502, 135.04367
16	Gallic acid	C_7_H_6_O_5_	171.99263 [M+H]+1	14.740	153.88593, 130.96609, 107.95045, 97.34501
17	Caffeic acid	C_9_H_8_O_4_	163.03850 [M+H-H_2_O]+1	4.403	135.04376, 117.03347, 89.03881

**Table 3:** Effect of AMP addition on the enzyme activity of honey. The results are presented as mean value ± standard deviation (SD; n = 3). Different lowercase letters indicate significant differences (*p* < 0.05).

Sample Name	Amylase (mL/g·h)	Glucose Oxidase (μg/g·0.5 h)	Sucrase (mg/g·h)
Original Honey	8.58 ± 0.15 ^a^	89.52 ± 2.69 ^a^	43.74 ± 2.70 ^a^
0.1% AMP Honey	7.74 ± 0.27 ^b^	83.12 ± 1.56 ^b^	41.84 ± 1.66 ^ab^
0.2% AMP Honey	7.11 ± 0.44 ^bc^	78.79 ± 2.19 ^b^	39.21 ± 1.72 ^bc^
0.3% AMP Honey	6.94 ± 0.35 ^c^	71.99 ± 1.32 ^c^	36.56 ± 1.43 ^cd^
0.4% AMP Honey	6.58 ± 0.31 ^c^	67.61 ± 2.34 ^c^	33.49 ± 1.47 ^de^
0.5% AMP Honey	5.21 ± 0.26 ^d^	57.81 ± 1.99 ^d^	29.47 ± 1.09 ^e^

**Table 4:** Original honey and 0.1~0.5% AMP honey solubility. Dissolution time and description of sample condition after dissolution. The results are expressed as mean ± standard deviation (n = 3). Different lowercase letters indicate significant differences (*p* < 0.05).

Sample Name	Solubility Time(s)	Solubility Situation
Original Honey	49.67 ± 2.08 ^d^	Clear and transparent, without impurities, with the smell of honey water.
0.1% AMP Honey	71.00 ± 2.65 ^ab^	Clear and transparent, with a very light pink color and no impurities. It has the scent of honey water and a very light berry aroma.
0.2% AMP Honey	66.67 ± 4.16 ^bc^	Clear and transparent, light pink, free of impurities, with the smell of honey water and a light berry smell.
0.3% AMP Honey	63.00 ± 3.61 ^c^	Clear, pink, free of impurities, with the smell of honey water and a certain berry smell.
0.4% AMP Honey	77.67 ± 1.15 ^a^	Clear and transparent, dark pink, without impurities, with the smell of honey water and an obvious berry smell.
0.5% AMP Honey	74.67 ± 6.81 ^a^	Clear and transparent, pink-purple, with trace amounts of undissolved AMP particles, with the smell of honey water and a strong berry smell.

## Data Availability

The original contributions presented in the study are included in the article, further inquiries can be directed to the corresponding author.
